# Prevalence and factors associated with VIA positive result among clients screened at Family Guidance Association of Ethiopia, south west area office, Jimma model clinic, Jimma, Ethiopia 2013: a cross-sectional study

**DOI:** 10.1186/s13104-015-1594-x

**Published:** 2015-10-29

**Authors:** Zewdie Mulissa Deksissa, Fessahaye Alemseged Tesfamichael, Henok Assefa Ferede

**Affiliations:** Columbia University, International Center for AIDS Care and Treatment Program, Addis Ababa office, P.O. Box 5566, Addis Ababa, Ethiopia; Department of Epidemiology, College of Public Health and Medical Sciences, Jimma University, P.O. Box 1274, Jimma, Ethiopia

**Keywords:** Cervical cancer, Visual inspection with acetic acid, VIA positive result, Cryotherapy

## Abstract

**Background:**

Cervical cancer is the 2nd most frequent and top killer cancer among women in Ethiopia. Prevalence and factors associated with visual inspection with acetic acid (VIA) positive result is not studied yet at the study area.

**Methods:**

A cross-sectional study was conducted at Jimma model clinic of Family Guidance Association of Ethiopia, from September 11, 2013 to October 11, 2013. Pertinent data of 334 screened clients were transferred to Epidata version3.1 using checklist, double data entry verification done and exported to SPSS version16.0. After cleaning the data, descriptive analysis was done and logistic regression model employed to identify predictors of VIA positive result. Statistical significance was declared at P < 0.05.

**Results:**

Out of 334 screened clients, 43 (12.9 %) had VIA positive result. Initiation of sexual intercourse earlier than 16 years was found to be an independent predictor increasing the risk of VIA positive by 2.2 times as compared to clients who started at the age of 16 or more years (AOR [95 % CI] = 2.2 [1.1, 4.3]).

**Conclusions:**

Early initiation of sexual intercourse was an independent predictor of VIA positive result in this study. Thus, any cervical cancer prevention and control effort at the study area should address the problem of early initiation of sexual intercourse.

**Electronic supplementary material:**

The online version of this article (doi:10.1186/s13104-015-1594-x) contains supplementary material, which is available to authorized users.

## Background

Cervical cancer is a disease in which the cells of the cervix become abnormal and start to grow uncontrollably, forming tumors [1]. It is caused by the sexually transmitted human papilloma virus (HPV) infection which has been detected in up to 99 % of women with squamous cervical carcinoma [2]. Young age at first intercourse (<16 years), multiple sexual partners, cigarette smoking and high parity are risk factors for HPV acquisition and cervical cancer.

Cancer of the cervix is the second most common cancer among women worldwide, with about 530,000 new patients diagnosed and over 270,000 deaths every year. It is a major cause of morbidity and mortality among women in low and middle income countries (LMICs) where more than 85 % of the global burden and deaths occur because of poor access to screening and treatment services [3, 4].

In Africa, according to most recent estimates, 80,400 women are diagnosed with cervical cancer every year, the second most frequent cancer. 50,300 die from the disease every year, the leading cause of cancer death. Rates vary substantially across regions, with the incidence and death rates in East Africa, the region Ethiopia belongs to, and West Africa five times as high as the rates in North Africa [5].

In Ethiopia, the annual number of new cervical cancer cases was 4648 and 3235 (69.6 %) die from the disease making it the 2nd most frequent and top killer cancer among women according to an estimate by International agency for research on cancer (IARC) [4]. However, the figures most likely under-represent actual number of cases and deaths, given the low level of awareness, cost, limited access to screening and treatment services and lack of a national cancer registry [6]. In order to address the problem, visual inspection with acetic acid (VIA) and cryotherapy for cervical cancer prevention among people living with HIV/AIDS (PLWHA) had been started in Ethiopia on September 2010 with collaborative effort of Pathfinder International, Federal Ministry of Health (FMOH) of Ethiopia and the Stanford University Program for International Reproductive Education and Services (SPIRES). However, studies on prevalence and factors associated with VIA positive were limited at the study area and this research was primarily designed to address that.

## Methods

Family Guidance Association of Ethiopia (FGAE) is one of the leading non-governmental providers of sexual and reproductive health (SRH) care in Ethiopia. FGAE has 20 medium SRH clinics and 27 youth centers across Ethiopia. This study was conducted at Jimma model clinic (JMC), one of the 20 medium SRH clinics of FGAE, Jimma town, 350 km southwest of Addis Ababa, Ethiopia’s capital. The catchment area of the clinic is Jimma town and surrounding districts. The clinic started opportunistic screening of females aged 25–45 years on September 2012 as per cervical cancer prevention guideline for low-resource settings [7]. Thus, after proper counseling of clients aged 25–45 years who came for medical or reproductive health services, those with free will were screened with 5 % acetic acid and test positive cryotherapy eligible clients were treated with cryotherapy while cryotherapy ineligible clients and those with lesions suspicious for cancer were referred to Jimma University specialized hospital (JUSH). Diagnostic criteria were as per cervical cancer prevention guideline for low-resource settings and screening results were defined as:

VIA positive: presence of raised and thickened white plaques or acetowhite epithelium, usually near the Squamo-columnar junction (SCJ).

VIA negative: presence of smooth, pink, uniform and featureless cervix; cervical ectropion; polyp; cervicitis; inflammation; and/or nabothian cyst after applying a dilute solution of acetic acid.

Eligible for cryotherapy: acetowhite lesion <75 % of cervix; lesion does not extend onto the vaginal wall; and lesion extends <2 mm beyond the diameter of the cryoprobe.

Ineligible for cryotherapy: acetowhite lesion >75 % of cervix; lesion extends into the vaginal wall; lesion extends >2 mm beyond the diameter of the cryotip and lesion suspicious for cancer.

Suspicious for cancer: presence of cauliflower-like growth or ulcer; fungating and bleeding mass.

Primary data was registered on standard client evaluation form for cervical cancer prevention service by trained general practioner and nurse. Ethical approval was obtained from ethical review board of Jimma University. A letter of support was obtained from JMC. Client records were treated confidentially and name of clients was not included in the data collection. After checking for integrity and plausibility, data was collected from standard client evaluation form for cervical cancer prevention service on checklist for retrieving data from September 11, 2013 to October 11, 2013 and transferred to Epidata. Double entry verification was also made and the entered data was exported to SPSS version 16.0 for analysis. Descriptive analysis of variables involved was done and Logistic regression was employed for identifying predictors of VIA positive result.

## Results

### Baseline characteristics of screened clients

A total of 334 clients aged 25–45 years were screened by (VIA) from September 2012 to October 11, 2013 at the study clinic. More than half (51.5 %) of them were in the age range 25–30 years with mean age of 32.4 (SD5.4) years. Most of them were married (73.7 %), multiparous (69.2 %), having only one sexual partner (73.1 %) and had no history of STI (84.7 %). More than half of them were HIV negative (51.2 %) and initiated sexual intercourse at 16 years or older (52.4 %) with mean age at initiation 16.7 (SD3) years. None of the screened clients had ever smoked, used steroid chronically or had abnormal Pap smear previously. Squamo-columnar junction (SCJ) was visible in all the screened clients (Table 1).

**Table 1:** Baseline characteristics of clients screened using VIA at Family Guidance Association of Ethiopia, south west area office, Jimma model clinic, Jimma, 2013

Characteristics	Number (%)
Age in years	
25–30	172 (51.5)
31–35	79 (23.7)
36–40	61 (18.3)
41–45	22 (6.6)
Educational status	
Illiterate	73 (21.9)
Primary	105 (31.4)
Secondary	105 (31.4)
Tertiary	51 (15.3)
Marital status	
Single	11 (21.9)
Married	246 (73.7)
Divorced	38 (11.4)
Widowed	23 (6.9)
Separated	16 (4.8)
Parity	
Nulliparous	31 (9.3)
Primiparous	72 (21.6)
Multiparous	231 (69.2)
Age at first intercourse	
<16	148 (44.3)
16	175 (52.4)
Unknown	11 (3.3)
Current contraceptive	
OCP	20 (6)
DEPO	57 (17.1)
Implanon	9 (2.7)
Jadelle	6 (1.8)
IUCD	6 (1.8)
Condom	7 (2.1)
BTL	2 (0.6)
Dual contraception	8 (1.5)
None	222 (66.5)
STI history	
Yes	51 (15.3)
No	283 (84.7)
HIV sero-status	
Unknown	28 (8.4)
Negative	171 (51.2)
Positive	135 (40.4)
HIV positives	
On HAART	110 (81.5)
Not on HAART	25 (18.5)
No of sexual partners	
One	244 (73.1)
Multiple	90 (26.9)
Yes	334 (100)
SCJ seen	
No	0

### Prevalence of VIA positive result

Of 334 screened clients, 43 (12.9 %) were found to have VIA positive result while 287 (85.9 %) had negative test result. The remaining four (1.2 %) were found to have lesions suspicious for cancer. Fourty-two of the 43 (97.7 %) clients with positive VIA test result were eligible for cryotherapy while one of the clients with positive VIA test result had lesion larger than cryoprobe by greater than 2 mm and thus not eligible. Thus, 42 clients with positive VIA test result who were eligible for cryotherapy were treated at the clinic while one of the clients who is ineligible for cryotherapy is referred to JUSH along with the four patients who had lesions suspicious for cancer (Table 2).

**Table 2:** VIA test result, cryotherapy eligibility and reasons for referral among clients screened at Family Guidance Association of Ethiopia, south west area office, Jimma model clinic, Jimma, 2013

Characteristics	Number (%)
VIA test result	
Positive	43 (12.9)
Negative	287 (85.9)
Suspicious for cancer	4 (1.2)
Cryotherapy eligibility	
Eligible	42 (97.7)
Ineligible	1 (2.3)
Reasons for referral	
Suspicious for cancer	4 (80)
Lesion larger than cryoprobe >2 mm	1 (20)

### Factors associated with VIA positive result

**Fig. 1:**
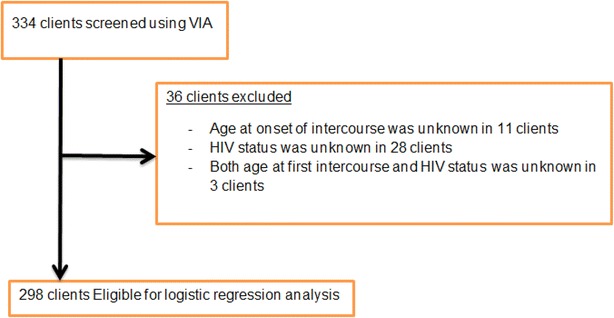
Profile of clients screened using VIA at Family Guidance Association of Ethiopia, south west area office, Jimma model clinic, Jimma, 2013. Three hundred and thirty-four clients were screened using VIA at Jimma model clinic. Age at onset of intercourse and HIV-status were unknown in 11 and 28 screened clients respectively while both age at onset of intercourse and HIV-status were unknown in 3 resulting in exclusion of a total of 36 screened clients from logistic regression analysis. Thus, only 298 were eligible for logistic regression analysis

Age at onset of intercourse and HIV-status were unknown in 11 and 28 screened clients respectively while both age at onset of intercourse and HIV-status was unknown in 3 resulting in exclusion of 36 screened clients from logistic regression analysis. Thus, of the 334 clients screened at the clinic, only 298 were eligible for logistic regression analysis (Fig. 1). Significant association was observed on bivariate logistic regression between VIA positive and age of clients and age at first intercourse. On multivariable logistic regression, clients who started intercourse at less than 16 years were 2.2 times (AOR [95 % CI] 2.2 [1.1, 4.3]) more likely to have VIA positive as compared to those who started intercourse at the age of 16 or more years (Table 3). However, history of STI, number of sexual partners, HIV-status and HAART-status were not found to be predictive of VIA positive in this study.

**Table 3:** Logistic regression analysis of factors associated with VIA positive at Family Guidance Association of Ethiopia, south west area office, Jimma model clinic, Jimma, 2013

Covariates	VIA result			
	Positive, no. (%)	Negative, no. (%)	COR [95 % CI]	AOR [95 % CI]
Age				
25–30	30 (17.8)	139 (82.2)	1	1
31–35	8 (10.1)	71 (89.9)	0.5 [0.2, 1.1]	0.5 [0.2, 1.2]
36–40	3 (5)	57 (95)	0.3 [0.1, 0.9]*	0.4 [0.1, 1.1]
41–45	2 (9.1)	20 (90.9)	0.4 [0.1, 1.9]	0.5 [0.1, 2.3]
Age at first intercourse				
16 years	17 (9.7)	158 (90.3)	1	1
<16 years	22 (15.3)	122 (84.7)	2 [1.1, 4.0]*	2.2 [1.1, 4.3]*
Current use contraceptive				
No	25 (11.4)	194 (88.6)	1	1
Yes	18 (16.2)	93 (83.8)	1.4 [0.8, 2.7]	1.7 [0.9, 3.3]
History of STI				
No	35 (12.5)	245 (87.5)	1	1
Yes	8 (16)	42 (84)	1.3 [0.6, 3.1]	1.6 [0.7, 3.6]
Number of sexual partners				
One	32 (13.3)	209 (86.7)	1	1
Multiple	11 (12.4)	78 (87.6)	0.9 [0.5, 2.9]	0.7 [0.3, 1.6]
HIV sero-status				
Negative	20 (11.9)	148 (88.1)	1	1
Positive	21 (15.7)	113 (84.3)	0.8 [0.4, 1.5]	1.2 [0.6, 2.6]
HIV positives				
Not on HAART	2 (8.3)	22 (91.7)	1	1
On HAART	19 (17.3)	91 (82.7)	0.7 [0.2, 2.4]	0.7 [0.2, 2.6]

## Discussion

Of 334 screened clients, 12.9 % had VIA positive and those who started sexual intercourse earlier than 16 years were 2.2 times at higher risk as compared to those who started sex at the age of 16 or more. The mean age at initiation of intercourse was 16.7 (SD = 3) years and this is comparable with median age at first intercourse for women aged 25–49 years both in Oromia (17 years) and Ethiopia (16.6 years) [8].

The prevalence of VIA positive at the study clinic (12.9 %) was similar to study finding from central Ethiopia among PLWHA’s (11 %) [9] but lower than study finding from southern Ethiopia among PLWHA’s (22.1 %) [10]. It was also similar to study finding from Madagascar (11.3 %) [11], Malawi (12.4 %) [11], Latin America (12 %) [12] and Thailand (13.3 %) [13] though it is lower than study findings from Nigeria (16 %) [14], Sudan (16 %) [15] and Bangladesh (18 %) [16]. However, it is higher than study findings from Uganda (7.8 %) [11] and Tanzania (9.7 %) [11]. The difference in prevalence could be due to the differences in the age of study populations [10], as evidenced by the two Ethiopian studies among PLWHA in which one used the age group 30–45 years [9] while the other used 18 years and older [10]. It may also be due to differences in test providers skills [17, 18] and underlying prevalence of other sexually transmitted infections [18]. The higher prevalence in Sudan [15], Nigeria [14] and Bangladesh [16] is most probably due to a lower sample size in the study, 100, 125 and 44 respectively, although provision of the service by laywomen, poor test providers skills, has also contributed to the finding of Bangladesh study. Further, VIA also inherently suffers from the same challenges as other visual interpretation methods including colposcopy and cytology as evidenced by Indian study where VIA positivity rate varied from 4 % to 31 % among the six gynecologists who performed the test [17].

Early initiation of intercourse increased the risk of VIA positive by 2.2 times which is similar to study from Brazil (OR [95 % CI] 1.97 [1.18–3.3]) [19], Nigeria (OR [95 % CI] 3.7 [1.07–12.8]) [14] and India (OR [95 % CI] 3.5 [1.1–10.9]) [20]. Early onset of sexual activity is thought to be associated with high risk because, during puberty, cervical tissue undergoes physiologic changes, transformation zone on the ectocervix is enlarged, and exposure to HPV at such times may facilitate infection which may make this area more vulnerable to development of dysplasia, a cervical squamous precancer [21]. Kenyan study has also showed higher risk of VIA positive among HIV patients (AOR [95 % CI] 4.8 [1.8–12.4]), those having multiple sexual partners (AOR [95 % CI] 3.8 [1.1–13.5]) [22] and HIV patients not on antiretroviral therapy (HAART) (AOR [95 % CI] 2.21 [1.28–3.83]) [23]. Tanzanian study showed higher risk of VIA positive among widowed/separated (OR [95 % CI]  1.41 [1.17–1.66]) and grand multipara women (OR [95 % CI] 3.19 [1.84–5.48]) [24]. South Ethiopian study reported higher risk among those with history of sexually transmitted disease (AOR [95 % CI] 2.30 [1.23, 4.29]) [10]. However, age of the client, history of STI, number of sexual partners, HIV-status and HAART-status were not found to be predictive of VIA positive in this study.

The role of HIV in precancerous cervical lesion is thought to be mediated through immune suppression [1]. Thus, prompt initiation of HAART through an early enrollment into care has an impact on reducing the prevalence and progression of cervical precancerous lesions [23]. Women who are separated or widowed may have higher number of lifetime sexual partners in comparison with married women and as number of lifetime sexual partners increases, the risk of HPV infection increases and thus they are more susceptible for developing precancerous lesions [25]. High parity increases the risk of precancerous cervical lesions most likely due to repeated cervical trauma during consecutive births and hormonal adjustment during and after pregnancies which may create an entry point for the HPV virus [26]. History of sexually transmitted disease increases the risk of precancerous cervical lesions due to the sexually transmitted nature of HPV infection [27].

## Conclusions

In this study, 12.9 % of screened clients had VIA positive and early initiation of intercourse was found to be an independent predictor increasing the risk by 2.2 times. Thus, there is a need to introduce HPV vaccination for girls aged 9–13 years, advocate for the norm of virginity till marriage, avoid early age at marriage, promote delaying of age at initiation of sexual intercourse, give sexuality education tailored to age and culture and promote and provide condom for those engaged in sexual activity in addressing early initiation of sexual intercourse (Additional files 1, 2).

## Additional files

10.1186/s13104-015-1594-x Standard Client Evaluation form for Cervical Cancer Prevention service.
10.1186/s13104-015-1594-x Family Guidance Association of Ethiopia (FGAE), Jimma model clinic, checklist for retrieving data from standard Client Evaluation form for Cervical Cancer Prevention service: September 2013.

## Abbreviations

AIDS: acquired immuno-deficiency syndrome
AOR: adjusted odds ratio
CI: confidence interval
COR: crude odds ratio
FGAE: Family Guidance Association of Ethiopia
FMOH: Federal Ministry of Health
HAART: highly active antiretroviral therapy
HIV: human immunodeficiency virus
HPV: human papilloma virus
JMC: Jimma model clinic
JUSH: Jimma University specialized hospital
LMICs: low and middle income countries
OR: odds ratio
PLWHA: people living with HIV/AIDS
SCJ: squamo-columnar junction
SD: standard deviation
SPIRES: Stanford University Program for International Reproductive Education and Services
STI: sexually transmitted infections
VIA: visual inspection with acetic acid
