# Supplementary material for: Prevalence and factors associated with VIA positive result among clients screened at Family Guidance Association of Ethiopia, south west area office, Jimma model clinic, Jimma, Ethiopia 2013: a cross-sectional study
Source: BMC Res Notes. 2015 Oct 29;8:618. doi: 10.1186/s13104-015-1594-x (PMC4627428; doi:10.1186/s13104-015-1594-x)
Supplement: Supplementary file 1 — 10.1186/s13104-015-1594-x Standard Client Evaluation form for Cervical Cancer Prevention service. [file 13104_2015_1594_MOESM1_ESM.docx]

**Additional file1**: **Standard Client Evaluation form for Cervical Cancer Prevention service**
