# Supplementary material for: Prevalence and factors associated with VIA positive result among clients screened at Family Guidance Association of Ethiopia, south west area office, Jimma model clinic, Jimma, Ethiopia 2013: a cross-sectional study
Source: BMC Res Notes. 2015 Oct 29;8:618. doi: 10.1186/s13104-015-1594-x (PMC4627428; doi:10.1186/s13104-015-1594-x)
Supplement: Supplementary file 2 — 10.1186/s13104-015-1594-x Family Guidance Association of Ethiopia (FGAE), Jimma model clinic, checklist for retrieving data from standard Client Evaluation form for Cervical Cancer Prevention service: September 2013. [file 13104_2015_1594_MOESM2_ESM.docx]

**Additional file2**: **Family Guidance Association of Ethiopia (FGAE), Jimma model clinic, Checklist for retrieving data from standard Client Evaluation form for Cervical Cancer Prevention service: September 2013.**

1. **CLIENT IDENTIFICATION**
2. MRN___________________________
3. VIA register No:_________________
4. Age______________
5. Educational status(enter last grade completed)________________
6. **REPRODUCTIVE HISTORY**:
7. Marital status: _______
8. parity: ________
9. current contraceptive(s)_______
10. age at first intercourse:_____
11. **RISK FACTORS**
12. Number of sexual partner(s)of Client: _______________ Of spouse_________
13. History of STI of Client: □Yes □NO of Partner: □Yes □No
14. HIV/AIDS testing: □Unknown □If tested, enter status: □Reactive □Non-reactive
15. If reactive, is the patient currently on HAART: □Yes □ No
16. History of smoking □Yes □NO
17. Previous abnormal pap smear □Yes □NO
18. Chronic corticosteroid use □Yes □NO
19. **PELVIC EXAMINATION**
20. SCJ was completely seen □Yes □No
21. **VIA RESULT**: □Suspicious for Cancer □Negative □Positive
22. **CRYOTHERAPY ELIGIBILITY**: □ eligible Ineligible
23. **REASON FOR REFERRAL:** □Suspicious for cancer □Lesion larger than cryoprobe>2mm

□Lesion>75% □Client denied cryotherapy

□ Lesion extended inside os

*Information recorded by--------------------------------- Date----------------------------*

*Approved by ------------------------------------------------ Date--------------------------------*
